# A Coordinated Wheeled Gas Pipeline Robot Chain System Based on Visible Light Relay Communication and Illuminance Assessment

**DOI:** 10.3390/s19102322

**Published:** 2019-05-20

**Authors:** Wen Zhao, Mitsuhiro Kamezaki, Kento Yoshida, Kaoru Yamaguchi, Minoru Konno, Akihiko Onuki, Shigeki Sugano

**Affiliations:** 1 Graduate School of Creative Science and Engineering, Waseda University, Tokyo 169-8050, Japan; kame-mitsu@aoni.waseda.jp (M.K.); k_yoshida@sugano.mech.waseda.ac.jp (K.Y.); k_yamaguchi@suganos.mech.waseda.ac.jp (K.Y.); sugano@waseda.jp (S.S.); 2 Tokyo Gas Co. Ltd., Tokyo 105-8527, Japan; m-konno@tokyo-gas.co.jp (M.K.); ak-onuki@tokyo-gas.co.jp (A.O.)

**Keywords:** visible light communication (VLC), pipeline robot, illuminance assessment, gas inspection

## Abstract

The gas pipeline requires regular inspection since the leakage brings damage to the stable gas supply. Compared to current detection methods such as destructive inspection, using pipeline robots has advantages including low cost and high efficiency. However, they have a limited inspection range in the complex pipe owing to restrictions by the cable friction or wireless signal attenuation. In our former study, to extend the inspection range, we proposed a robot chain system based on wireless relay communication (WRC). However, some drawbacks still remain such as imprecision of evaluation based on received signal strength indication (RSSI), large data error ratio, and loss of signals. In this article, we thus propose a new approach based on visible light relay communication (VLRC) and illuminance assessment. This method enables robots to communicate by the ‘light signal relay’, which has advantages in good communication quality, less attenuation, and high precision in the pipe. To ensure the stability of VLRC, the illuminance-based evaluation method is adopted due to higher stability than the wireless-based approach. As a preliminary evaluation, several tests about signal waveform, communication quality, and coordinated movement were conducted. The results indicate that the proposed system can extend the inspection range with less data error ratio and more stable communication.

## 1. Introduction

Japan owns, in total, a 200,000 km gas pipeline distribution network. However, it is a country with frequent severe natural disasters such as floods and earthquakes. Gas pipeline networks need to be inspected and maintained regularly since the leakage caused by destruction such as corrosion, deformation, and cracking will endanger the safe supply of gas. In recent years, various pipeline robots for pipe inspection and maintenance have been developed as they are more effective and cost less compared with traditional destructive pipe inspection methods. Destructive inspection is inefficient because it requires the pipeline to be removed from the ground. Besides, pipeline robots are more precise and time-saving than conventional external inspection technologies based on non-destructive sensor network inspection technologies such as fiber optic distributed temperature sensing (DTS) system [1].

The application of pipeline robot for inspection and maintenance has been regarded as one of the most suitable and effective solutions available. The inspection system usually consists of a robot which performs the inspection and transfers the data back to the controller terminal using a cable or wireless device. There are many kinds of approaches being proposed for inspecting pipelines including a CCTV camera, penetrating radar, ultrasonic sensor, infrared thermography, pressure (water, gas, etc.) sensor, concentration (gas) detection sensor, in-pipe temperature sensor, etc. After the information from the pipe is collected by these sensors, it requires transmitting to the external terminal in real time. Besides, most robots are manually controlled by operators through the control terminal and the control signals should be also transmitted efficiently. Therefore, the inspection robots require a stable, efficient, real-time, long-distance, and high-quality information interaction with the external terminal. Currently, it is still difficult for robots to realize the above requirements because of communication problems within the pipe. Whether a wired or wireless pipe inspection robot, they are limited by the complicated pipe environment. This limitation is mainly manifested in the cable restriction and wireless attenuation.

In this article, we propose a new pipe inspection robot chain system (RCS) based on visible light relay communication and illuminance assessment. The proposed system has several advantages. (1) It can effectively increase the inspection range with the RCS due to the wireless signal relay between multiple robots. (2) The visible light relay communication (VLRC) enables the robots to achieve high-quality and low-loss communication with the external controller. (3) The visible light communication (VLC) system can not only achieve distance estimation and communication but also provide illumination for pipeline inspection. 

## 2. Related Works

Several studies related to pipeline inspection robots have been carried out. ALSTOM and MRINSPECT are two typical wired robots with cables of Ethernet and RS485. In a straight pipe, the standard communication distance can reach about 50–80 m at 100 kbit/s-5 Mbit/s transmission rate. In complex pipeline environments, the inspection distance can be significantly reduced due to the communication cable’s restriction such as friction with elbow and entanglement [2,3,4]. Another effective communication approach in pipe inspection is using wireless communication. The most well-known robots are EXPLORER and KANTARO, which have a flexible inspection system. The 2.4 GHz Wi-Fi/ IEEE 802.11b technology is adopted in these robots. The transmission rate can reach almost 2 Mbit/s, but the effective communication distance is limited to 30–50 m. This distance can be shorter if they are deployed in a metal pipe with small diameter (e.g., 50 mm) [5,6,7,8]. Moreover, they have an inspection system based on a wireless sensor network (WSN). In the system, an underground, in-pipe robot serves as a sensor node (SN) that captures and transmits information of the pipe. An above-ground sensor network functions as a relay node (RN) for signal processing and relaying. Such a system has the potential to extend the communication distance. However, most pipes are deeply buried to avoid underground facilities, hence wireless signals cannot pass through thick soil layers or underground facilities, and therefore, this system can only be applied to shallow pipes [9].

Currently, most pipeline inspection robots still have several limitations, which can be mainly classified into two factors: environmental and technical factors. The environmental factors are that the wireless signal attenuation can degrade the communication quality and reduce the inspection range of the robot. The reason is that when the wireless signal is transmitted in the pipe, the huge energy loss can occur due to the reflection and refraction of microwaves. Besides, the same problems exist in the inspection method based on robot-sensor hybrid networks, such as WSN. As stated above, wireless signals are unable to pass through the thick soil layers or underground facilities, therefore the wireless communication is also restricted. In addition, due to the unexpected environment complexities (i.e., elbows and T-junctions) in the pipe, the restrictions from communication cables in the pipe can be considered as a technical factor for wired robots.

In our former research, on the basis of the analysis of arbitrary pipeline environments, we have proposed a tracked pipe inspection robot chain system based on 2.4 GHz wireless relay communication. This system contains one ‘leader robot’ for in-pipe inspection and several ‘follower robots’ for the signal relay. To ensure the stability of this relay communication between adjacent robots, we have adopted a RSSI-based evaluation method for coordinated movement of RCS [10]. Moreover, a wireless application layer communication protocol (WALCP) is used to enhance the stable performance of wireless relay communication. This system can enlarge the inspection range and provide some advantages compared with WSN-based inspection systems, such as higher applicability to the more complex environment regardless of pipeline layout and its depth. However, from the experimental results, we reveal some defects as follows. (1) The wireless signals possess severe attenuation when they are transmitted, especially in the small diameter metal pipe. (2) The communication quality is also not good since the data error ratio (DER) and data loss ratio (DLR) increase significantly when the transmission rate rises. (3) Since the wireless signal is not stable in the pipe, RSSI-based distance estimation and communication become inaccurate with floating and inaccurate RSSI data. These features can increase the risk of relay communication failure [11]. 

In response to these problems, as a preliminary study, we propose a new RCS based on the VLRC. Recently, VLC technology has received attention since it can increase the available bandwidth for wireless communication which uses visible light (band: 380 nm~80 nm) to transmit information. Since the concept of VLC was proposed in 2001, many related researches have promoted the development of this technology. In 2008, Little et al. developed a point-to-point bidirectional data transmission system using white LEDs as a light source. This system adopts the OOK modulation method which can reach a maximum 56 kbps transmission rate [12]. In 2009, Zeng et al. proposed an optical multiple input multiple output (MIMO) technology based on white LED array and detector array. Then they analyzed the data transmission capabilities of this MIMO system. The maximum transmission rate could reach about 1 Gbps [13]. In 2013, Azhar et al. proposed a MIMO system which adopts white light source. The system adopts orthogonal frequency division multiplexing (OFDM) technology to realize indoor communication with 1 Gbit/s transmission rate [14]. 

In our research, visible light can also be used as a communication medium due to its high-speed encoding and transmitting features and an illumination function for pipe inspection. Compared with a wireless signal, VLC owns a high communication quality, especially the low transmission error ratio. Due to the short wavelength and diffuse reflection in the pipe, light owns less attenuation in the pipe, especially in the small diameter pipe. An illuminance assessment method for distance estimation and stable communication can work more precisely and effectively than the RSSI-based method. In summary, this system can not only extend the inspection range but also overcome the uncertainty and instability of wireless signal attenuation and can finally realize a precise and stable communication in the pipe.

## 3. Analysis of VLRC Transmission Channel

In this study, we first analyze the concept of the VLRC channel. The VLRC system can be described as a multi-path transmission system and is composed of several baseband linear and time-invariant subsystems. Each subsystem owns instantaneous input power of the emitted light $P_{i}\left(t\right)$ and the output current $I\left(t\right)$ after photoelectric conversion. The relationship between these parameters can be summarized by the following equation [15,16,17]:

(1) $$I\left(t\right)=\eta P_{i}\left(t\right)⊗h\left(t\right)+N\left(t\right),$$

where η represents the photosensitivity of the photo-detector, $h\left(t\right)$ indicates the impulse response of channels, and $N\left(t\right)$ denotes the white additive Gaussian noise [18]. 

The $\left(n-1\right)\;rd$ average transmitted optical power $P_{t}\left(n-1\right)$ can be given by:

(2) $$P_{t}\left(n-1\right)=\underset{T\to \infty }{\lim}\int_{-T}^{T}P_{i}\left(t\right)dt,$$

The n rd average received optical power $P_{r}\left(n\right)$ can be determined by:

(3) $$P_{r}\left(n\right)=H\left(0\right)P_{t}\left(n-1\right),$$

where $H\left(0\right)$ is the direct current channel gain which can be described as $H\left(0\right)=\int_{-\infty }^{\infty }h\left(t\right)dt$, n represents the number of VLC relay node.

**Figure 1:**
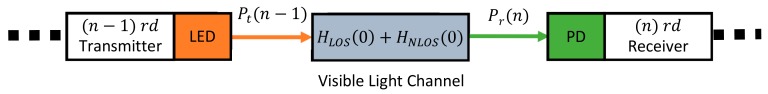
Channel structure of visible light relay communication (VLRC).

Here, we consider that the transmission channel of visible light in the pipe includes two links: the unobstructed line-of-sight (LOS) links and non-line-of-sight (NLOS) links [19,20,21]. LOS links rely on a direct transmission path between the transmitter and receiver for communication, whereas NLOS links usually rely on diffuse reflection from the pipe wall. During the transmission in the pipe, both LOS and NLOS should be considered. The VLRC channel structure is shown in Figure 1. The received power is generally described by the channel direct current gain of LOS and reflected path of NLOS as the following equation:

(4) $$P_{r}\left(n\right)=H_{LOS}\left(0\right)P_{t}\left(n-1\right)+H_{NLOS}\left(0\right)P_{t}\left(n-1\right).$$

## 4. Hardware Development of System

In this section, a fundamental wheeled RCS is explained. A relay communication mechanism based on VLC is adopted in such a system. The RCS mainly consists of three communication nodes: a controller terminal, a follower robot which acts as a ‘signal relay node’, and a leader robot which mainly undertakes pipeline inspection tasks. The detailed communication system will be introduced in Section 5.

### 4.1. System Architecture

**Figure 2:**
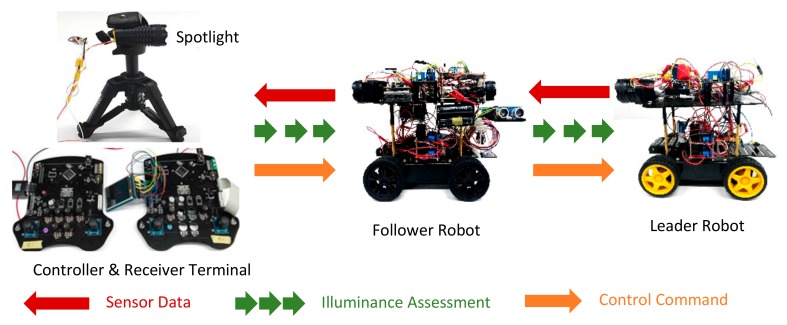
Whole architecture of the robot chain system.

**Figure 3:**
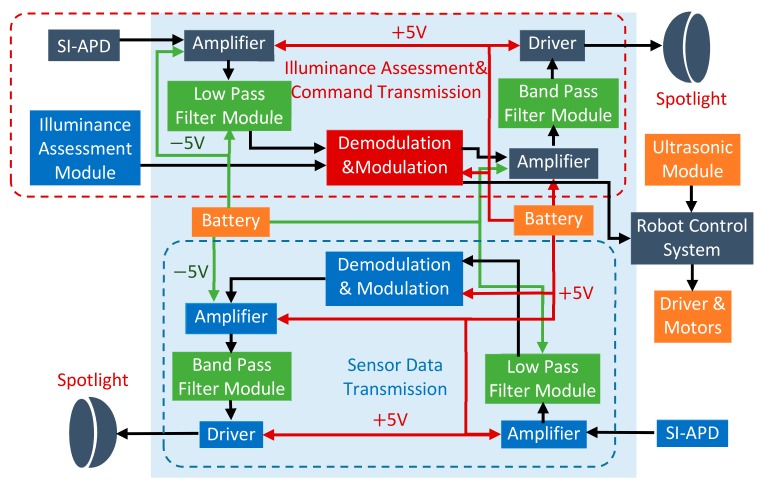
The electrical structure of the follower robot.

**Figure 4:**
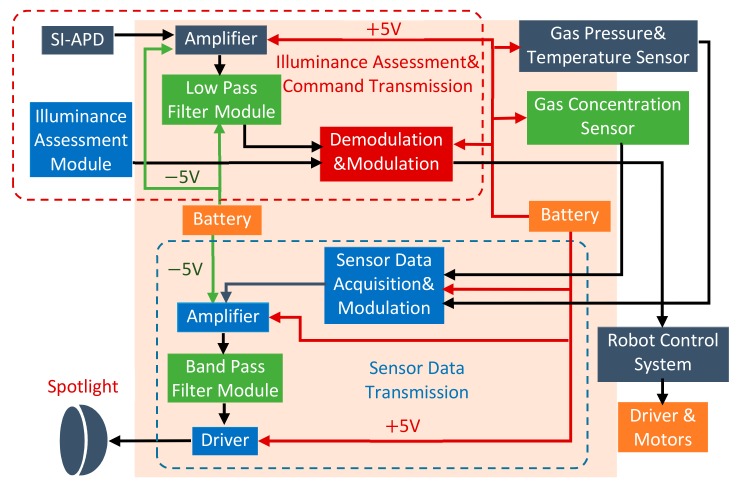
The electrical structure of the leader robot.

The RCS can be conceptualized as the following major functional blocks with the corresponding hardware architecture shown in Figure 2. Figure 3 and Figure 4 mainly describe the main mechanical and electrical mechanism in the leader and follower robots. This system consists of:

- **Control terminal:** This functional block is a micro-controller based on STM32F407VGT. It deals with the following tasks: (1) collecting analog control signals or commands and converting these signals to digital signals for robots, (2) modulating digital signals with pulse width modulation (PWM) method, (3) signal amplifying, filtering, (4) driving the light emitting diode (LED) spotlight.
- **Receive terminal:** This functional block is responsible for the reception and processing of sensor data. It directly handles visible light with the silicon PIN photodiode (SI-PIN PD). Then, it converts the signals into electrical signals by using a PIN PD. After signal amplifying, filtering, and demodulating, the sensor data from the leader robot are restored.
- **Leader robot:** This robot plays the most important role in the whole system. It is capable of completing gas pipe inspection tasks with gas detection sensors. The robot contains three parts: (1) The gas detection and transmission module: The robot can collect the information inside the pipe with barometric gas pressure/temperature sensor BMP280 and gas concentration sensor CCS811. The signals are processed and transmitted through the ‘relay node’ in the form of visible light. (2) The visible light intensity indication assessment module: The robot can realize the coordinated movement and reliable VLRC with the support of this module. The detail functions will be explained in Section 4.3. (3) The visible light receiver, motor drive, and robot controller modules: The robot can be controlled by the relay visible light signal from the control terminal.
- **Follower robot:** As a relay node, it can receive different kinds of data and retransmit to the corresponding object (leader or receiving terminal). It adopts a special VLC application protocol to prevent information conflict and mutual interference. This mechanism will be described in Section 5.2. The follower robot can achieve self-navigation in the pipe with three ultrasonic sensors.

### 4.2. VLC Transmitter Design

**Figure 5:**
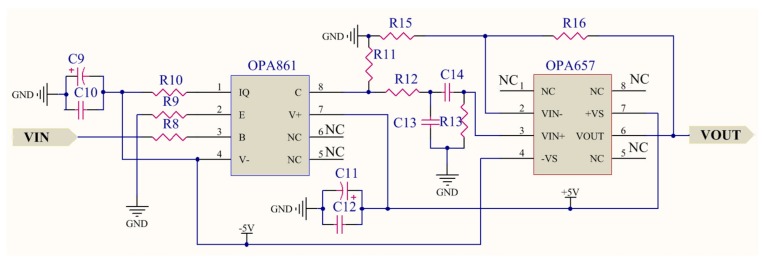
Schematic design of the signal amplifier and filter of a visible light communication (VLC) transmitter.

**Figure 6:**
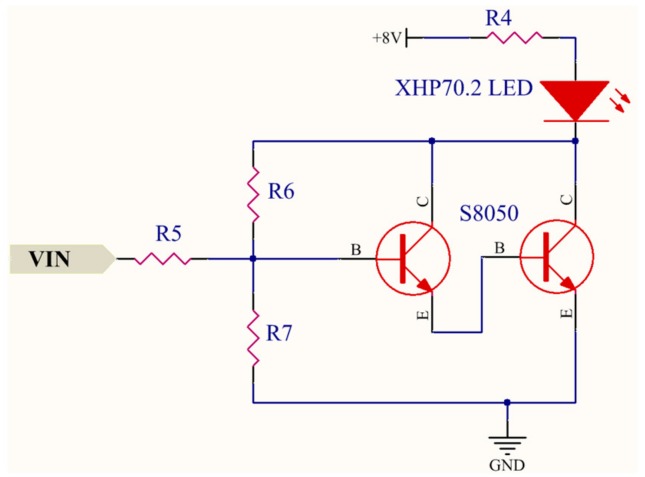
Schematic design of the LED driver.

To establish the duplex VLRC, each robot and control terminal have VLC transmitters. In the control terminal, the functions of the transmitter realizes are signal analog/digital (A/D) conversion, signal modulation, signal amplifying, filtering, and wave transmission. The robot does not have A/D converters (ADCs), but the other functions are almost the same as the control terminal. In these transmitters, A/D conversion and signal modulation are completed in ARM-based STM32F407ZGT6 micro-controller. This module has several 12 bit ADCs, which are successive approximation ADCs [22,23,24]. In this research, two-channel analog signals of joystick controller are captured and converted into 12-bit binary digital data. These digital signals can be modulated through the PWM modulation method. The modulation process is explained in Section 5 in detail. Figure 5 shows the schematic design of the signal amplifier and filter. The signals from the micro control unit (MCU) are firstly amplified by the OPA861 [25,26]. This chip provides a high dynamic range amplifier for wide-band transimpedance amplifier applications. It also owns high bandwidth of approximately 80 MHz. A second-order passive band-pass filter based on OPA657 is applied to filter out the low- and high-frequency components which can affect the normal signal transmission [27]. As the most indispensable part of the VLC system, wave transmission module loads the modulated electrical signal onto the LEDs and converts electrical signals into optical signals. Since the current output of the main control ARM chip is too small, the modulated signal has to be amplified to display the change in LED optical power [28,29]. As illustrated in the LED driver of Figure 6, we selected two NPN S8050 transistors as the Darlington amplifier. The maximum collector current of S8050 can reach 1500 mA. The current gain is set to about 110. It can almost satisfy the distortion-free transmission of modulated signals and power supply requirements of the LED light. The characteristics of LEDs have a profound impact on the communication quality and distance of the entire VLC system. Generally, white LEDs own a great advantage in light sources. We thus select XLamp XHP 70.2 LEDs provided by CREE. Co. as a light source for this RCS. Such LED owns 4292 l m output and maximum drive current can reach (Standard) 4.8 A (6 V) and 2.4 A (12 V) [30]. 

### 4.3. VLC Receiver and Illuminance Assessment Module

**Figure 7:**
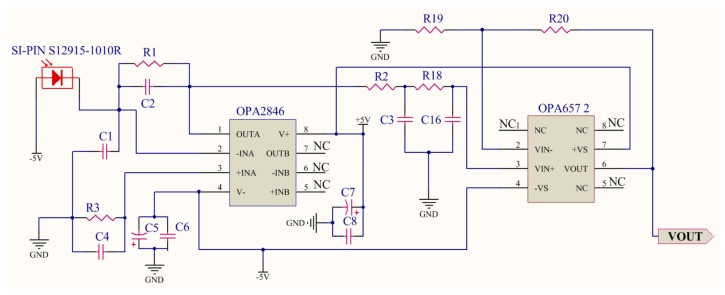
Schematic design of signal amplifier and filter of VLC receiver.

The main function of the VLC receiver is to convert the optical signal to the electrical signal. In the receiver, the PD detects the optical signal, and at the same time, converts it into a current signal. After the amplifier processing and demodulation, the control signal can be restored. As the core part of the receiver, PD can convert optical wave energy into a current signal. Considering visible light wavelength range from 380–780 nm, in this research, we select SI-PIN PD S12915-33R which is provided by Hamamatsu Co. The maximum photoelectric conversion efficiency of such a product can reach almost 0.64 A/W. The photosensitive area is about 5.76 ${\mathrm{mm}}^{2}$, the spectral response range is from 340 nm to 1100 nm, and the response frequency can reach maximum of 60 MHz [31]. Similar to wireless transmission, the optical signal attenuation exists in the open air and also there are many interferences and noise issues during the channel transmission, so in the receiver part, amplifier and filter circuit for further pre-processing of the optical signal are important. We thus choose OPA2846 and OPA657 as an amplifier and high-frequency filter processor, respectively. OPA2846 is a low-noise, wide-band, typical dual, and voltage-operational amplifier. When setting up OPA2846 as the broadband PD amplifier, the important elements in the design is the diode capacitance (C1) with the reverse bias voltage (−5 V) and the desired transimpedance gain (R1). The schematic design is shown in Figure 7. It illustrates a design using a 10 pF source capacitance diode and a 10 kΩ transimpedance gain. It is necessary to adopt the low pass filter in the receiver due to ambient light such as sunlight in the open air. The frequency of light is much higher than the carrier signal we adopt for light communication, so these high-frequency components have to be filtered out in the process of filtering. As the filter circuit described in Figure 7, the OPA657 chip is used to build a second-order low pass filter circuit.

The digital illuminance sensor BH1750FVI is installed around the photodiode module. BH1750FVI is a digital ambient light sensor for I2C bus interface [32]. This module can capture from weak ambient light (1 lx) to strong light (65528 lx). It fits to be used as the illuminance assessment for light relay communication. In the later experiments, we determine the precise illuminance parameter for the RCS. Using BH1750FVI, we have also divided the VLC into three regions: good communication (1165 lx < illuminance< 1495 lx), poor communication (974 lx < illuminance < 1165 lx), and lost communication (illuminance<974 lx). In the first region, each robot is in good communication with each other. In the second region, each robot’s function is abnormal because of poor VLC quality. In the last region, each robot may lose communication with each other. In the coordinated movement test, the robots function based on this illuminance assessment. 

## 5. Software Development of System

The software developed in this study contains modulation/demodulation between two robots and relay light communication of the whole system.

### 5.1. Modulation and Demodulation

For the VLC system, a good selection of modulation and demodulation approach can obviously reduce multipath interference, and finally improve communication efficiency. The most important parts of this research are the technology of optical signal intensity modulation and demodulation. In the design of VLC system, the coding technology of communication is generally based on On Off Key (OOK), Pulse Position Modulation (PPM), Binary Phase Shift Keying (BPSK), and Orthogonal Frequency Division Multiplexing (OFDM). Based on the analysis of these types of modulation approaches, OOK is the simplest modulation method, however, the transmission rate is very low with huge signal transmission delay [33]. BPSK method can effectively cut down inter-symbol interference in wireless channels, whereas the transmission information ability and bandwidth utilization rate are very low [34]. As some of the important modulation methods used in VLC systems, PPM and OFDM methods have a high transmission rate. However, they have a low anti-interference ability and high DER to external light [35,36]. This means the VLC system can be applied to the environment without ambient light. 

In this article, we adopt a PWM method for the VLC system because it is easier to realize by hardware and has a relatively low DER at high transmission rate. This method can transform binary information code into pulse width waves through micro-controller, and then the LED drive module transmits a pulse width optical signal to the transmission channel. The advantages of this modulation approach lie in its strong anti-noise capability and high transmission rate. By adjusting the pulse width, information can be loaded into the output waveform. These pulses have the same amplitude and different widths. The digital signals ‘1’ and ‘0’ are defined based on the pulse width in one period. The carrier frequency used is set to 2~2.68 KHz. When transmitting a signal ‘1’, it takes 500 μs, including 200 μs low level and 300 μs high level. When sending ‘0’, it also requires 350 μs, including 200 μs low level and 150 μs high level. In the receiver, the signal can be demodulated by determining the pulse width of the received pulse signal. For example, in our VLC control system, we choose the Grove-thumb Joystick controllers. There are two 0–10 kΩ potentiometers for X-axis and Y-axis respectively which controls 2D movement of the robot by analog signals. When the module is in working mode, the output will be two kinds of analog values, representing X- and Y-axis directions. The micro-controller can detect these two analog values and convert into 12 bits digital signal through two successive approximation ADCs [37]. 

At the receiver, the TIM1 input pin of MCU is directly connected to the PD module. As an advanced control timer, TIM1 can be easily set to the input capture mode. The special capture/compare register (TIM1_CCRx) is used to detect and measure the square wave pulse width. Then, we demodulate the detected signal according to the transmission protocol used by the signal modulation module. When entering the ID detection interruption program, the system receives and detects the pulse signal [38]. After passing the ID checking, the ID detection flag is set to ‘1’, and the ID detection interruption finishes and enters into the program of data detection interrupt. Here the digital signal ‘1’ or ‘0’ is determined according to the pulse width calculation in one period. After completing receiving 12 bits data of control command and detecting the end mark, they will convert 12-bit binary data to a decimal control command for robot control. 

### 5.2. VLRC Mechanism

In the relay transmission, the modulation/demodulation mechanism is the same as direct transmission between the controller terminal and one robot. Here we adopt a VLRC mechanism for duplex communication in the RCS. The phenomenon of scattering and diffuse reflection exists when the light is transmitted into the pipe [39,40,41]. This phenomenon has an impact on light-based communication quality. For the above reason, a mechanism based on the message identifier is presented in this study. This mechanism is capable of preventing information conflict and mutual-interference from light scattering and diffuse reflection, and thus can improve the security and accuracy of information transmission [42]. 

#### 5.2.1. Message Identifier Structure and Frame Format

The VLRC mechanism is adopted in the design of the information frame. As mentioned in the modulation and demodulation section, each information frame contains: the message identifier, data of information, and end mark. The node ID of frame identifier is set as follows: one 100 μs, 120 μs, and 130 μs pulse width square wave which represent nodes: controller, follower, and leader robot, respectively. There are two types of signals in this communication system: control command and sensor data, so after setting of ‘node ID’, the ‘signal type ID’ is set as follows: two 100 μs and two 125 μs pulse width square waves indicate sensor data and control command, respectively. Then before data transmission, there are two 100 μs square waves required for starting transmitting the data frame. The transmission of 12 bits digital data requires 7.2 ms in total [43]. After the transmission of 12 bits data, there is one square pulse with a width of 200 μs as the end mark. This mechanism enables the identification of useful message and filters out the useless message the receiver receives.

#### 5.2.2. Software Realization of Relay Process

**Figure 8:**
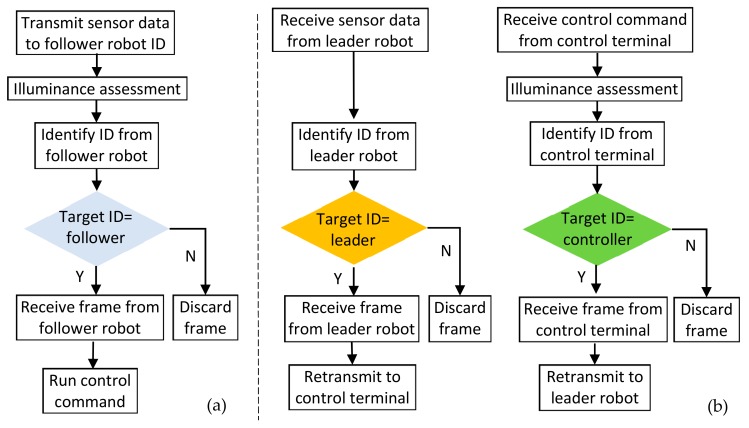
Relay frame routing of (**a**) leader robot and (**b**) follower robot.

As in Figure 8, relay frame routing follows this principle: The leader robot is the terminal receiver, which only receives data frames from the control terminal over the relay of the follower robot. Moreover, the leader robot can identify the control command ID and further process these command signals. This leader robot can also send sensor data frame with the ID identifier to the follower robot. As the function of the follower robot is ‘relay node’, it can receive the sensor information with corresponding ID from the leader robot and bypass this message to the control terminal. Similarly, it can bypass the control command from the control terminal to the leader robot with the corresponding ID [44]. 

## 6. System Performance Evaluation

To verify the performance of the proposed RCS, a series of experiments were carried out. (1) The waveform tests of VLRC between the transmitter, follower robot, and leader robot were conducted in the laboratory environment. (2) The two parameters of the RCS: DER and DLR were measured at different distances and different transmission rates. (3) When performing the in-pipe experiments, the effective communication region was determined with the leader robot in a 300 mm diameter straight pipe. (4) When testing the coordinated movement of the RCS, illuminance assessment, an assessment of the light illuminance power present in a received light signal, was recorded. We also tested the coordinated movement of the RCS in the pipe with steel cover to avoid the ambient light, and illuminance was recorded during this experiment [45]. 

### 6.1. Waveform Test of System

**Figure 9:**
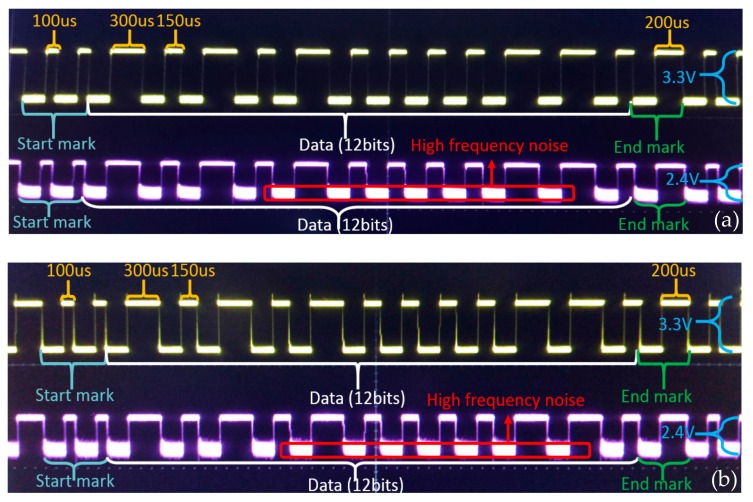
The waveform of transmission between (**a**) control terminal and follower robot (**b**) follower robot and leader robot.

In the laboratory environment, we first conducted VLRC waveform testing on control command signals. The interval between each adjacent robot was 1 m. To avoid the ambient light, we turned off the room light. As illustrated in Figure 9a, the entire data frame of the control command was recorded. This signal indicated the signals sent by the control terminal. The yellow waveform was composed of multiple rectangular wave signals. The purple waveform represented the signals received by the ‘relay node’ of the follower robot. The high-frequency noise was added to the transmission channel in the purple waveform. The effective voltage of signal changed from 3.3 V to 2.4 V [46]. Figure 9b demonstrated the signal waveforms transmitted between the follower and leader robot. The yellow waveform indicated the signal transmitted by the ‘relay node’. In ‘relay node’, this signal was re-modulated and amplified from the light signal. The purple wave denoted the actual light signal that the leader robot received after the second channel attenuation in the open air. Although some signals had a certain attenuation and some a high-frequency noise, this relay communication could still satisfy the further experimental requirements and realize the effective light transmission. 

### 6.2. Communication Quality Test of System

**Figure 10:**
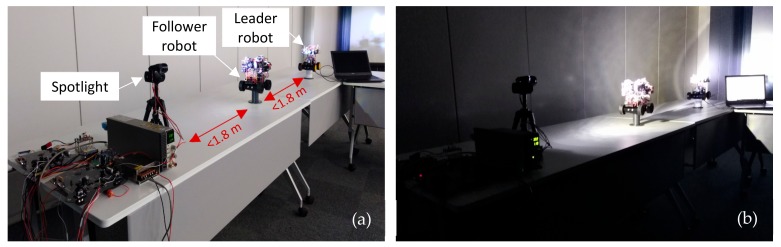
Experimental setting for communication quality testing.

**Figure 11:**
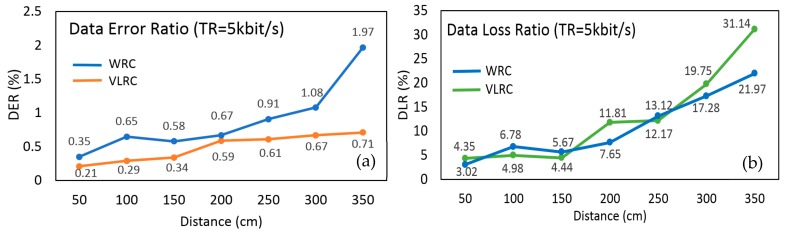
(**a**) Data error ratio (DER) and (**b**) Data loss ratio (DLR) test at different distance.

**Figure 12:**
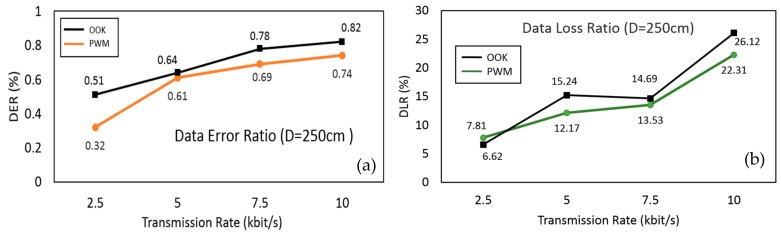
(**a**) DER and (**b**) DLR test at different transmission rate.

To further investigate the reliability of the RCS, a series of VLRC quality test were carried out in the lab. Two important parameters: DER and DLR were measured and analyzed [47]. Figure 10 depicts the experiment setting for this test. The distance between each robot was about 180 cm. The maximum total relay communication distance between the leader robot and the controller can reach about 360 cm (2 × 180 cm). The control terminal continuously sent a standard 12 bits frame data 0xFFF to the ‘relay node’ of the follower robot at a certain transmission rate. The leader robot received this data through this ‘relay node’ and the statistics of the received data at the leader robot were evaluated in this communication quality test. DER means the error rate the data frame received by the leader robot compared to the standard frame sent by the control terminal. DLR indicates the percentage of the data frame that is lost during light transmission or demodulation. To measure these parameters in a dark environment under different data rates and different distance can help us to estimate the maximum transmission distance and suitable transmission rate. First, in this system, we adopted the 2.5 kbps transmission rate and tested DER and DLR at different distances from 50 to 350 cm (maximum 360 cm) [48]. As described in Figure 11a,b, the DER and DLR increased as distance extended. When the transmission distance of the system was within 150 cm, the DER and DLR could reach less than nearly 0.5% and 5%, respectively. When the distance was more than 150 cm, both parameters rose significantly. The reason for this is that the longer the transmission distance, the more obvious the scattering effect of light, and the greater the transmission attenuation in the channel. We tested the communication quality of RCS at 250 cm distance using four different transmission rates: 2.5, 5, 7.5, and 10 kbit/s [49]. As shown in the analysis from Figure 12a,b, notably from 2.5 kbit/s to 10 kbit/s, the DER just increased from 0.32% to 0.74%, while the DLR rose significantly from 7.81% to 22.31% with PWM method. Through these experiments, one conclusion can be obtained: that the higher the transmission rate, the higher the chance of data transmission loss and error. From Figure 12a,b, OOK method was evaluated as a reference standard to compare with PWM in order to show that the PWM method had relatively low DER at the same transmission rate. The OOK method showed larger data error than the PWM method when the transmission rate was set below 10 kbit/s. As a special Amplitude Shift Keying (ASK) method, OOK method was based on carrier amplitude modulation. It was the most energy-saving method because it transmitted energy only when digital data "1" was sent. However, it could only demodulate the signal with a high signal-to-noise ratio. Therefore compared with PWM, it required for high hardware demodulation ability. Big DLR occurred because of failure of demodulation. Although OOK was much easier to realize, the transmitter using the OOK method had low transmitting power. In the same transmission distance, the DLR and DER were much larger than the PWM method. Whereas PWM had many advantages such as quick response time and lower DER at a rate below 10 kbit/s, we therefore used the PWM method for our system. Figure 11a also depicted that DER of WRC rose from 0.35% to 1.97%, which was higher than VLC (0.21–0.71%). In Figure 11b, the DLR of WRC and VLRC were almost the same except for the longer distance such as 350 cm. At this distance, the VLRC system had bigger DLR = 31.14% than WRC because of huge light scattering in transmission. In this research, DER was more important for information transmission in the pipe inspection mission. 

### 6.3. Experiment on Relationship between Illuminance Assessment and Communication Region

**Figure 13:**
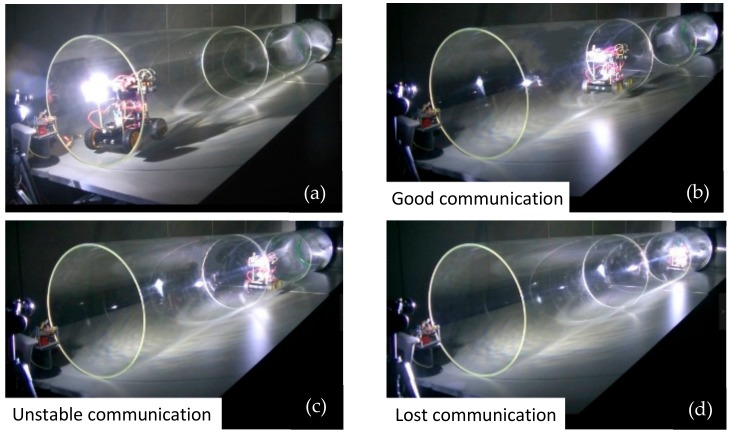
Movement test with one leader robot.

**Figure 14:**
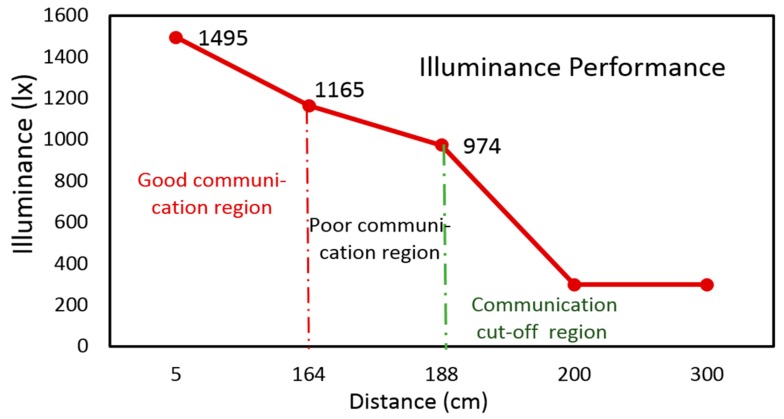
Test on illuminance performance of leader robot.

Before analyzing the comprehensive performance on the RCS based on VLRC, the ‘light-control’ quality and precision of a single robot should be considered. In Figure 13, the leader robot was placed in the 3.5 m length pipe. The illuminance of control terminal spotlight was about 1500 lx. Through the tests, three zones (good, poor and communication cut-off) could be divided. From Figure 13 and Figure 14, the robot was initially in a good communication region (DER< 0.45%) [50]. When the robot entered the pipe to a depth of approximately 164 cm, the illuminance reduced to 1165 lx. Meanwhile, it was in a poor communication state. Some decode error of control command and severe data frame loss was observed. The robot might function abnormally in this region of poor visible light quality. As illustrated in Figure 14, the distance of 164–188 cm was considered as the poor communication region. Above 188 cm, the robot lost communication with the illuminance of 974 lx [51]. Through these tests, the relationship between illuminance and effective communication region could be determined. 

### 6.4. Movement and Illuminance Assessment Test in the Transparent Straight Pipe 

**Figure 15:**
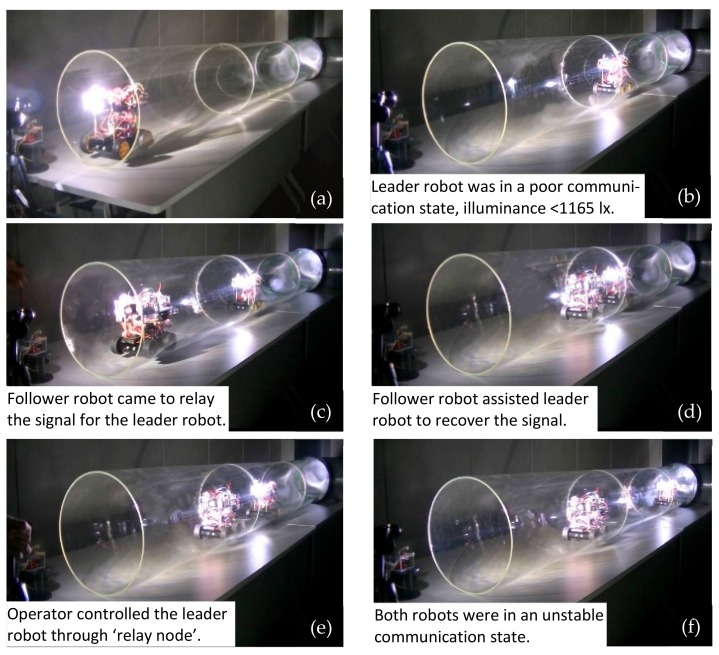
Coordinated movement of Robot Chain System (RCS) in transparent pipe.

The movement tests of the RCS were conducted in the 3.6 m length transparent pipe. Figure 15 illustrated the coordinated movement of the whole system in the pipe. In this test, the leader robot was designed to conduct a mission of pipe inspection with detection modules. It could obtain pipe information such as air pressure, temperature and gas concentration. This information was processed and sent by light carrier to the follower robot. The leader robot was manually controlled through the control terminal. It first entered the pipe. When it arrived at the region where the communication was unstable, it stopped and waited for the follower robot to recover the light signal. Then, the leader robot could communicate again with the control terminal with the support from ‘relay node’.

**Figure 16:**
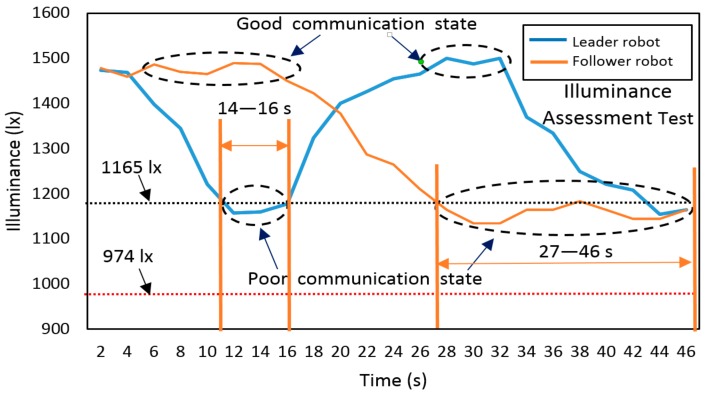
Comprehensive illuminance assessment test of RCS in the transparent pipe.

In this test, an important parameter about the illuminance of both ‘leader robot’ and ‘follower robot’ was also recorded and illustrated in Figure 16. The illuminance assessment could reflect the strength of the visible light signal received by each robot of the system. Entirely based on the preliminary measurement on a single robot, we could get the conclusion that each robot could keep a good ‘link’ through VLC when only the illuminance was above the 1165 lx. A black dotted line was added to the figure to indicate a stable and unstable communication boundary line with illuminance = 1165 lx. Moreover, a red dotted line was added to show the threshold of losing communication with illuminance = 974 lx. The whole experimental mission took nearly 46 s. The leader robot first entered the pipe at 2 s and moved forward to the target detection region. At around 14–16 s, the leader robot reached the region of ‘poor communication’, and illuminance decreased significantly from about 1495 lx to 1165 lx. According to the setting of the program, the robot stopped at this moment. Then, we placed the follower robot in the pipe, and it could move to the leader robot based on the self-navigation mode. Before 18 s, the follower one was at the pipe entrance with a good ‘link’ with the control terminal. The follower one could keep stable communication with the controller until 27 s. After this time, it was at the boundary of poor communication mode, which might have resulted in control error or sensor data error. However, during the period from 16 to 27 s, the leader robot recovered the communication with the help of ‘signal relay’ from the follower one. At the end of this test, the system was at the boundary of good communication conditions with stable illuminance of approximately 1165 lx. 

### 6.5. Movement and Illuminance Assessment Test in the Straight Pipe with Steel Cover

**Figure 17:**
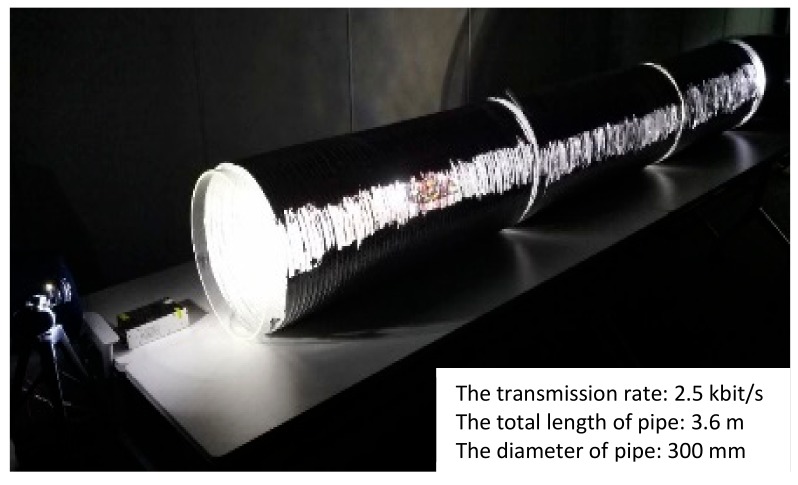
Coordinated movement of RCS in the pipe with steel cover.

**Figure 18:**
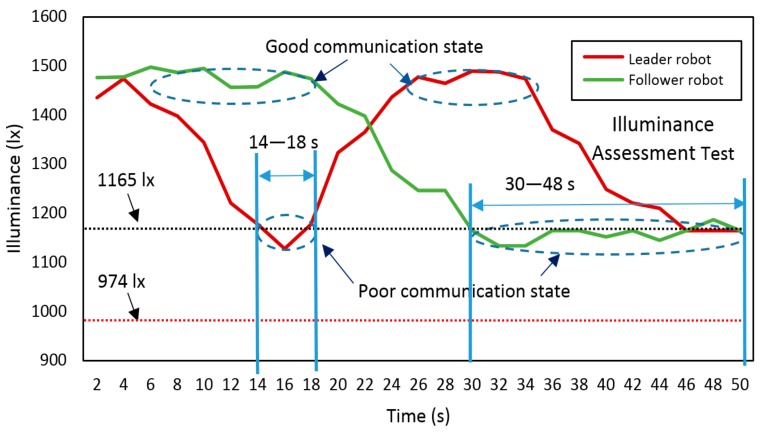
Comprehensive illuminance assessment test of RCS in pipe with steel cover.

To further verify the comprehensive performance of such a system, the movement tests in the transparent pipe are not enough. There are two reasons: (1) Since visible light can penetrate the pipe wall and create a big energy loss. (2) The ambient light can also affect the experimental results. Thus, we built a simulated environment with a steel cover to reduce these effects. Figure 17 and Figure 18 illustrated the coordinated movement of RCS in the pipe with steel cover. The RCS was operated to complete the whole mission including the ‘signal relay’ and coordinated movement as experiments before. The illuminance was measured and recorded during the movement test. As is similar with tests in the transparent pipe, the communication between leader robot and control terminal became unstable communication region at about 14 s and soon recovered the signal with the ‘signal relay’ from follower robot at around 18 s. When the follower robot entered the pipe at 30 s, it could be near the boundary of unstable communication with the control terminal. However, the leader robot was in a good communication region because of the ‘signal support’ from the follower one. In Figure 18, the leader robot was in a poor communication state at 14 s, while at 11 s in Figure 16. Before losing signal in the poor communication state, the leader robot in the steel pipe travelled a longer distance than in the transparent pipe. By further analyzing the experimental results, we could find that due to the light diffuse reflection in the pipe, the light energy attenuation was smaller than in the transparent pipe. This resulted in the longer transmission range in the pipe. 

**Figure 19:**
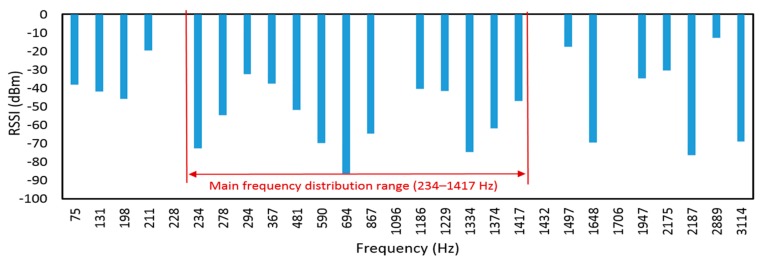
Spectral analysis of RSSI for the leader robot in the pipe with steel cover.

**Figure 20:**
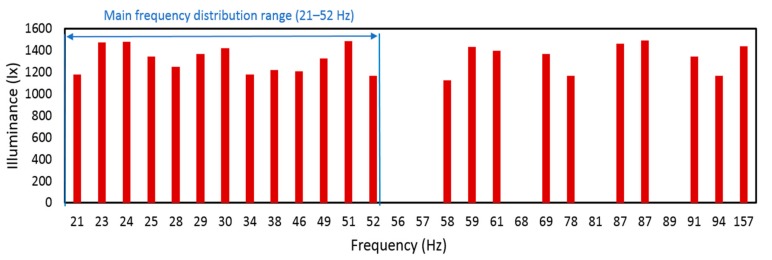
Spectral analysis of illuminance for the leader robot in the pipe with steel cover.

Finally, we took the leader robot as an example and made a spectrum analysis of the former RSSI-based and proposed the illuminance-based evaluation method. These two signals were converted into frequency domain signals by FFT transformation. Figure 19 was obtained based on the analysis of the former wireless-based RCS [10]. From Figure 19 and Figure 20, we could see that the main frequency distribution range was from 234 to 1417 Hz, whereas the frequency range of illuminance from illuminance-based RCS was from 21 to 52 Hz. Since RSSI was highly affected by reflections by the pipe wall, the high-frequency noise exists. These noises would affect the evaluation method and generate imprecise results. In this research, the illuminance-based assessment method for the RCS was more stable and precise than the former RSSI-based approach since it owned less high-frequency noise. 

## 7. Conclusions and Future Work

In this study, in order to overcome the challenge of limited inspection range brought by cable friction and wireless attenuation, a preliminary study using visible light communication was conducted, then a wheeled robot chain system based on the VLRC was developed. To verify the feasibility and performance of this system, we carried out four tests. Firstly, waveform experiment of relay signal transmission was carried out in the laboratory environment. The results revealed that such a relay communication system could realize the effective light transmission with low noise. Secondly, the system was tested on communication quality. Although VLRC possessed bigger DLR than wireless communication, the DER was smaller. DER was more important for information transmission in the pipe inspection mission. Thirdly, an experiment on the relationship between illuminance indication and communication region was conducted. Through this test, the effective communication region was determined. Finally, a set of movement tests were carried out in the transparent pipe and the pipe with steel cover. The results revealed that the coordinated movement of this system could be realized with VLRC and illuminance assessment. The illuminance assessment method was more precise than the RSSI-based evaluation method. 

Although the proposed system made breakthroughs, the defects of the system can also be found. These defects are highlighted below:

- In order to extend the inspection range with more effective and high-quality communication method, some VLC characteristics in the pipe should be studied further. These studies may contain: signal to noise ratio analysis, research on diffuse path gain of light in different pipelines, experiment on the effect of inter-symbol interference, study on diffuse reflection and scattering features of light transmission in gas medium, test on the influence of visible light components of different frequencies and wavelengths on light communication.
- The selection of PWM method in this research has not been appropriately justified. There is still a lack of numerical analysis to verify the transmission performance improvement based on such a method. Actually, the DER indicates that the PWM is a poor choice at low lux level. The redesign of the modulation/demodulation module and the relay module will be a better solution. From Figure 12a,b, the results demonstrate that if the transmission rate is beyond 10 kbps, big DER will occur. In addition, we have done the communication experiment at a transmission rate of 15 kbit/s for both methods. The results depict that the communication cannot be realized by both the PWM and OOK methods. Through the short distance test, we discovered that the demodulation system based on embedded STM32 we developed has limitation to capture and recognize the digital pulse accurately if the rate is beyond 10 kbit/s. Besides, a new MCU such as FPGA for modulation and demodulation should also be considered. Like the prototype, although this transmission rate is enough for robot controlling, it is not enough for video transmission for pipe inspection. The selection of other advanced modulation techniques should be considered and performance comparison at a high data rate and low lux level should also be implemented.
- This system can increase the risk of the system’s control and communication complexity. In order to make the system more robust and stable, a new control and communication algorithm should be further investigated.
- Such a system still has difficulties to adapt to a more complex pipe environment such as the elbow, T-junction, etc. The solution can be described as: (1) optimizing the mechanical structure of the robot, (2) improving communication ability in these complicated regions by analyzing and improving the property of light diffusion in elbow or T-junction.

## Abbreviations

The following abbreviations are used in this manuscript:

DTS	Distributed Temperature Sensing
WRC	Wireless Relay Communication
VLC	Visible Light Communication
VLRC	Visible Light Relay Communication
MIMO	Multiple Input Multiple Output
ADC	Analog-to-Digital Converter
MCU	Micro Controller Unit
OOK	On Off Key
BPSK	Binary Phase Shift Keying
PPM	Pulse Position Modulation
OFDM	Orthogonal Frequency Division Multiplexing
PWM	Pulse Width Modulation
RCS	Robot Chain System
DER	Data Error Ratio
DLR	Data Loss Ratio
RSSI	Received Signal Strength Indication
LED	Light Emitting Diode
WSN	Wireless Sensor Network
LOS	Line Of Sight
NLOS	Non Line Of Sight
WALCP	Wireless Application Layer Communication Protocol
RN	Relay Node
SN	Sensor Node

## References

[1] Sun X.G., Li J., Burgess D.T., Hines M., Zhu B.Y. A multicore optical fiber for distributed sensing. Proceedings of the SPIE 9098, Fiber Optic Sensors and Applications XI, 90980W.

[2] Roh S., Kim D.W., Lee J.S., Moon H., Choi H.R. Modularized in-pipe robot capable of selective navigation inside of pipelines. Proceedings of the IEEE/RSJ International Conference on Intelligent Robots and Systems (IROS).

[3] Lee D.H., Moon H., Choi H.R. Autonomous navigation of in-pipe working robot in unknown pipeline environment. Proceedings of the IEEE International Conference on Robotics and Automation (ICRA).

[4] Kim H.M., Suh J.S., Choi Y.S., Trong T.D., Moon H., Koo J., Ryew S., Choi H.R. An in-pipe robot with multi-axial differential gear mechanism. Proceedings of the IEEE/RSJ International Conference on Intelligent Robots and Systems (IROS).

[5] Schempf H., Mutschler E., Gavaert A., Skoptsov G., Crowley W. (2010). Visual and nondestructive evaluation inspection of live gas mains using the Explorer TM family of pipe robots. J. Field Robot..

[6] Ahrary A., Kawamura Y., Ishikawa M. A laser scanner for landmark detection with the sewer inspection robot KANTARO. Proceedings of the IEEE/SMC International Conference on System of Systems Engineering (ICSSE).

[7] Nassiraei A.A.F., Kawamura Y., Ahrary A., Mikuriya Y., Ishii K. Concept and design of a fully autonomous sewer pipe inspection mobile robot “KANTARO”. Proceedings of the IEEE International Conference on Robotics and Automation (ICRA).

[8] Ahrary A., Ishikawa M., Okada M. Experimental evaluation of intelligent fault detection system for inspection of sewer pipes. Proceedings of the IEEE/RSJ International Conference on Intelligent Robots and Systems (IROS).

[9] Wu D., Chatzigeorgiou D., Toumi Y.K., Mansour R.B. (2016). Node localization in robotic sensor networks for pipeline inspection. Trans. Ind. Inform..

[10] Zhao W., Kamezaki M., Yoshida K., Konno M., Onuki A., Sugano S. An automatic tracked robot chain system for gas pipeline inspection and maintenance based on wireless relay communication. Proceedings of the IEEE/RSJ International Conference in Intelligent Robots and Systems (IROS).

[11] Zhao W., Kamezaki M., Yoshida K., Konno M., Toriumi R., Sugano S. A reliable communication and localization method for gas pipeline robot chain based on RSSI theory. Proceedings of the IEEE/SICE International Symposium on System Integration (SII).

[12] Little T.D.C., Dib P., Shah K., Barraford N., Gallagher B. Using LED lighting for ubiquitous indoor wireless networking. Proceedings of the IEEE International Conference on Wireless and Mobile Computing, Networking and Communications.

[13] Zeng L.B., O’Brien D.C., Minh H.L., Faulkner G.E., Lee K. (2009). High data rate multiple input multiple output (MIMO) optical wireless communications using white led lighting. J. Sel. Areas Commun..

[14] Azhar A.H., Tran T.A., O’Brien D. (2013). A gigabit/s indoor wireless transmission using MIMO-OFDM visible-light communications. J. Photonics Technol. Lett..

[15] Pham Q.N., Rachim V.P., An J.Y., Chung W.Y. (2017). Ambient light rejection using a novel average voltage tracking in visible light communication system. Appl. Sci..

[16] Ciro R.A.M., Giraldo F.E.L., Perez A.F.B., Rivera M.L. (2018). Characterization of light-to-frequency converter for visible light communication systems. Electronics.

[17] Uddin M.S., Cha J.S., Kim J.Y., Jang Y.M. (2011). Mitigation technique for receiver performance variation of multi-color channels in visible light communication. Sensors.

[18] Xu Y.F., Zhao J.Q., Shi J.Y., Chi N. (2016). Reversed three-dimensional visible light indoor positioning utilizing annular receivers with multi-photodiodes. Sensors.

[19] Bosch C.L., Gonzalez I.A., Rodriguez D.S., Casanas C.R. (2016). Evaluation of the effects of hidden node problems in IEEE 802.15.7 uplink performance. Sensors.

[20] Pesek P., Zvanovec S., Chvojka P., Bhatnagar M.R., Ghassemlooy Z., Saxena P. (2018). Mobile user connectivity in relay-assisted visible light communications. Sensors.

[21] Tao S., Yu H., Li Q., Bai X., Tang Y. (2018). Power allocation of non-orthogonal multiple access based on dynamic user priority for indoor OOS-guaranteed visible light communication networks. Appl. Sci..

[22] Colado C.V., Arredondo B., Torres J.C., Fraguas E.L., Vergaz R., Martin D.M., Pozo G., Romero B., Apilo P., Quintana X. (2018). An all-organic flexible visible light communication system. Sensors.

[23] Carrascal C., Demirkol I., Paradells J. (2016). On-demand sensor node wake-up using solar panels and visible light communication. Sensors.

[24] Rodríguez J., Lamar D.G., Aller D.G., Miaja P.F., Sebastian J. (2018). Efficient visible light communication transmitters based on switching-mode dc-dc converters. Sensors.

[25] (2013). TI OPA861, Wide Bandwidth Operational Transconductance Amplifier (OTA), datasheet (Rev. G), SBOS338G–August 2005–rev. May 2013.

[26] Munoz P., Mico G., Bru L.A., Pastor D., Perez D., Domenech J.D., Fernandez J., Banos R., Gargallo B., Alemany R. (2017). Silicon nitride photonic integration platforms for visible, near-infrared and mid-infrared applications. Sensors.

[27] (2015). TI OPA657, 1.6-GHz, Low-Noise, FET-Input Operational Amplifier, datasheet (Rev. F), SBOS197F–December 2001–rev. August 2015.

[28] Pai K.J., Qin L.D., Lin C.H., Tang S.Y. (2018). Start-up current spike mitigation of high-power laser Diode driving controller for vehicle headlamp applications. Appl. Sci..

[29] Fuada S., Putra A.P., Aska Y., Adiono T. Trans-impedance amplifier (TIA) design for visible light communication (VLC) using commercially available OP-AMP. Proceedings of the Information Technology, Computer, and Electrical Engineering (ICTTACEE).

[30] (2017–2018). Cree XLamp XHP70.2 LEDs, Product Family Data Sheet, CLD-DS170 Rev 0F.

[31] (2018). Si Photodiodes, S12915 Series, For Visible to IR, General-Purpose Photometry, Datasheet, Cat. No. KSPD1086E02 September 2018.

[32] (2011). BH1750FVI, Digital 16bit Serial Output Type Ambient Light Sensor IC, Technical Note No. 11046EDT01, 2011.11—Rev. D.

[33] Zhang D.F., Zhu Y.J., Zhang Y.Y. (2013). Multi-LED phase-shifted OOK modulation based visible light communication systems. IEEE Lett. Photonics Technol..

[34] Shinwasusin E., Chalie C., Suksompong P. Modulation performance for visible light communications. Proceedings of the IEEE International Conference Information & Communications Technology for Embedded Systems.

[35] Afgani M.Z., Haas H., Elgala H., Knipp D. Visible light communication using OFDM. Proceedings of the IEEE International Conference on Testbeds & Research Infrastructures for the Development of Networks & Communities.

[36] Gancarz J.E., Elgala H., Little T.D.C. (2015). Overlapping PPM for band-limited visible light communication and dimming. IEEE Trans. Solid State Lighting.

[37] Zhan P., Yu K., Swindlehurst A.L. (2011). Wireless relay communications with unmanned aerial vehicles: Performance and optimization. IEEE Trans. Aerosp. Electron. Syst..

[38] Cetin O., Zagli I., Yilmaz G. (2013). Establishing obstacle and collision free communication relay for UAVs with artificial potential fields. J. Intell. Robot. Syst..

[39] Wang J., Al-Kinani A., Zhang W.S., Wang C.X., Zhou L. (2018). A general channel model for visible light communications in underground mines. J. Commun. Theor. Syst..

[40] Ding J., Xu Z. Performance of indoor VLC and illumination under multiple reflections. Proceedings of the IEEE International Conference on Wireless Communications and Signal Processing (WCSP).

[41] Mmbaga P.F., Thompson J., Haas H. (2016). Performance analysis of indoor diffuse VLC MIMO channels using angular diversity detectors. J. Lightwave Technol..

[42] Singh D., Sood A., Thakur G., Arora N., Kumar A. Design and implementation of wireless communication system for toll collection using LIFI. Proceedings of the IEEE International Conference on Signal Processing, Computing and Control (ISPCC).

[43] Niaz M.T., Imdad F., Kim H.S. (2017). Power consumption efficiency evaluation of multi-user full-duplex visible light communication systems for smart home technologies. Energies.

[44] Garcia G.C., Ruiz I.L., Nieto M.A.G. (2016). State of the art, trends and future of Bluetooth low energy, near field communication and visible light communication in the development of smart cities. Sensors.

[45] Li Z., Feng L., Yang A. (2017). Fusion based on visible light positioning and inertial navigation using extended kalman filters. Sensors.

[46] Masini B.M., Zanella A. (2018). A survey on the roadmap to mandate on board connectivity and enable V2V-based vehicular sensor networks. Sensors.

[47] Masini B.M., Bazzi A., Zanella A. (2018). Vehicular visible light networks for urban mobile crowd sensing. Sensors.

[48] Ji R., Wang S., Liu Q., Lu W. (2018). High-speed visible light communications: Enabling technologies and state of the art. Appl. Sci..

[49] Arredondo B., Romero B., Pena J.M.S., Pacheco A.F., Alonso E., Vergaz R., Dios C.D. (2013). Visible light communication system using an organic bulk heterojunction photodetector. Sensors.

[50] Huynh P., Yoo M. (2016). VLC-based positioning system for an indoor environment using an image sensor and an accelerometer sensor. Sensors.

[51] Do T.H., Yoo M. (2016). An in-depth survey of visible light communication based positioning systems. Sensors.

